# Clinical governance implementation in a selected teaching emergency department: a systems approach

**DOI:** 10.1186/1748-5908-7-84

**Published:** 2012-09-10

**Authors:** Ali Heyrani, Mohammadreza Maleki, Ahmad Barati Marnani, Hamid Ravaghi, Mojtaba Sedaghat, Mosadegh Jabbari, Davood Farsi, Abdoljavad Khajavi, Zhaleh Abdi

**Affiliations:** 1 Department of Health Services Management, School of Health Management and Information Sciences, Tehran University of Medical Sciences, Tehran, Iran; 2 Community Medicine Department, School of Medicine, Tehran University of Medical Sciences, Tehran, Iran; 3 Internal Medicine (Nephrology) Department, Hazrat Rasoul Akram Hospital, Tehran University of Medical Sciences, Tehran, Iran; 4 Emergency Medicine Department, Hazrat Rasoul Akram Hospital, Tehran University of Medical Sciences, Tehran, Iran

**Keywords:** Clinical governance, Emergency department, Implementation, Quality improvement, Soft Systems Methodology

## Abstract

**Background:**

Clinical governance (CG) is among the different frameworks proposed to improve the quality of healthcare. Iran, like many other countries, has put healthcare quality improvement in its top health policy priorities. In November 2009, implementation of CG became a task for all hospitals across the country. However, it has been a challenge to clarify the notion of CG and the way to implement it in Iran. The purpose of this action research study is to understand how CG can be defined and implemented in a selected teaching emergency department (ED).

**Methods/design:**

We will use Soft Systems Methodology for both designing the study and inquiring into its content. As we considered a complex problem situation regarding the quality of care in the selected ED, we initially conceptualized CG as a cyclic set of purposeful activities designed to explore the situation and find relevant changes to improve the quality of care. Then, implementation of CG will conceptually be to carry out that set of purposeful activities. The activities will be about: understanding the situation and finding out relevant issues concerning the quality of care; exploring different stakeholders’ views and ideas about the situation and how it can be improved; and defining actions to improve the quality of care through structured debates and development of accommodations among stakeholders. We will flexibly use qualitative methods of data collection and analysis in the course of the study. To ensure the study rigor, we will use different strategies.

**Discussion:**

Successful implementation of CG, like other quality improvement frameworks, requires special consideration of underlying complexities. We believe that addressing the complex situation and reflections on involvement in this action research will make it possible to understand the concept of CG and its implementation in the selected setting. By describing the context and executed flexible methods of implementation, the results of this study would contribute to the development of implementation science and be employed by boards and executives governing other clinical settings to facilitate CG implementation.

## Background

### The challenge of quality improvement in complex healthcare organizations

It has been noted that the quality of healthcare cannot be defined precisely [1]. As Donabedian hypothesized, a complex network of contingent and interacting factors make the concept of quality in healthcare [2]. Different definitions for healthcare quality and its dimensions have been issued in the related literature, based on diverse perspectives [3-6]. It is also generally accepted that the definition varies among policy makers, managers, professionals, and patients [7-9]. However, a number of models and methodologies have been introduced for suggesting initiatives, managing changes, and improving the quality of healthcare [10-12]. Despite similarities among the quality improvement models for healthcare organizations [13], various unsuccessful implementation attempts have been reported [14,15].

Furthermore, healthcare organizations are complex systems, and much of their complexity is believed to be due to the presence of a diverse spectrum of staff and departments working in a hierarchical non-flexible structure wherein several professional groups with different objectives, activities, and subcultures provide healthcare services [16]. Therefore, a successful quality improvement method in a setting may fail in other settings due to different underlying factors [17]. Despite these challenges, the quality of healthcare remains as one of the most important worldwide concerns [18]. Many countries have considered the improvement of hospitals’ performance as a major policy agenda for their health system [19].

### The introduction of clinical governance

In 1997, clinical governance (CG) was first introduced in England as a framework for improving quality of healthcare [20]. The England’s Department of Health defined CG as ‘a framework through which NHS organizations are accountable for continuously improving the quality of their services and safeguarding high standards of care by creating an environment in which excellence in clinical care will flourish’ [21]. Since then, in order to clarify the concept of the new framework, various health professions and disciplines have provided different definitions for CG and its core elements [22]. However, the ‘temple-like’ model of CG that comprises seven pillars and five substantial foundations seems to be the fundamental CG paradigm [23]. The famous seven pillars of CG—that have evolved somehow during the first years of the introduction of CG—are clinical effectiveness, clinical audit, risk management, patient and public involvement, staff and staff management, education and training, and use of information [23-25]. These pillars have been shown to be founded on five essential cornerstones that are: systems awareness, leadership, ownership, teamwork, and communication [23]. It is also widely accepted that an effective leadership is essential to drive cultural, structural, and systems change in order to guarantee successful implementation of CG [26,27].

During the past years, a few countries have started to apply different definitions and strategies of CG in their health systems [28-30]. In 2009, the World Health Organization Regional Committee for the Eastern Mediterranean strongly advocated that member states use frameworks such as CG to assess and enhance the quality of their hospital services [19].

### The challenge of CG implementation in Iran

Iran has strongly emphasized the provision of the high quality care in hospitals as one of the main goals of the country’s health system [31-34]. In this regard, there have been numerous attempts to apply quality improvement programs, such as Total Quality Management (TQM), clinical guideline implementation, internal audit, and staff education and training in Iranian hospitals [35,36]. In November 2009, the Ministry Of Health and Medical Education (MOHME) regarded the previous experiences with quality improvement programs as ‘valuable’ but with problems, especially those associated with ‘diversity of committees established for quality improvement,’ ‘fragmentation of quality improvement processes,’ and ‘lack of a holistic view that led to dispersed operations.’ Then, CG was introduced as the accepted framework to improve quality of hospital care in Iran [Iranian health minister’s official letter No.388044, November 2009 (Persian)]. Afterwards, the MOHME issued the ‘seven pillars’ model of CG (previously mentioned) and required all hospitals across the country to plan and provide necessary infrastructures to implement CG in accordance with that model [37].

The implementation of the new framework has not been a straightforward process for Iranian hospitals. During the past two years, the MOHME has repeatedly highlighted the important issues, such as serious determination, consistent actions, holistic view, staff education, and quality culture development for successful implementation of CG in hospitals [the MOHME official letters No.45025, 92561, 106083, and 113205 (Persian)]. Nonetheless, our preliminary interviews with hospital executives and professionals showed that they believed ‘ongoing processes are not aligned with quality improvement’ in their hospitals [AH, Personal communications, workshop series on ‘essentials of clinical governance,’ different provinces of Iran, October 2010 to June 2011]. Additionally, it seemed that almost all of these key people had not well understood the concept of CG and the appropriate approaches to implement it.

In a one-day seminar on ‘operational strategies for implementation of CG in Iran,’ held by the MOHME, it was suggested that ‘ambiguity of ‘quality’ in health services,’ ‘complex interactions among different players in the health system,’ ‘satisfaction of patients and their involvement in designing and monitoring care,’ ‘allocation of resources,’ and ‘applying effective tools and techniques in the various steps of the implementation’ were among the most challenging strategic issues in the implementation of CG [unpublished seminar, Tehran, July 2010]. Experts at the Office of Hospital Management and Clinical Excellence (at MOHME) regard CG as a holistic approach to improve patient-centeredness and clinical dimensions of quality. However, they think that despite the available knowledge about other countries’ experiences in CG, a comprehensive study is required to clarify the CG concept and its implementation steps, as well as different roles and responsibilities in implementation scheme in Iran [AH, Personal communications, 2011].

### Systems approaches

It is claimed that ‘systems approaches’ have emerged and evolved in response to uncertainty, diversity of problems, change, and increasing complexity in organizations [38,39]. All systems approaches claim to be holistic in nature, but they differ in underlying assumptions, methodologies, and practical methods [38].

‘Hard’ systems approaches assume a situation as ‘a system’ with an agreed-upon framework and goals [40]. They try to build models in order to understand the system’s structure and interconnections [38]. Using these models, it would be possible to suggest interventions to optimize the system and improve the situation [38]. In contrast, ‘soft’ systems approaches perceive a situation far more complex and problematic than it can be realized as a distinct ‘system’ [40]. They consider various worldviews, values, and objectives in a ‘problem situation’ which is often related to social and cultural phenomena [40]. Soft systems approaches believe that a hasty attempt to frame a problem situation as ‘a system’ and an early application of optimizing models can result in a distortion of the real situation [38]. Instead, these approaches use their models to inquire into a situation, learn from inquiring, and make accommodations between different players to improve the situation [40]. Some authors consider a third type of systems approaches [39]. While holding almost similar assumptions to soft approaches, ‘critical’ systems approaches specially consider ‘power relations’ in problem situations [39]. It is then believed that hard systems approaches are often used for solving clear and specified technical problems, where soft systems approaches are appropriate for improving complex and ill-defined situations that require social and cultural considerations [38-40].

### Study purpose and objectives

The purpose of this study is to understand how CG can be defined and implemented in an emergency department (ED). We considered an ED as our study setting, because these departments are believed to be complex environments that play an essential role in hospital care systems and need a special attention regarding quality improvement [41,42]. Also, in Iran, the improvement of performance and quality of care in EDs has received great importance, so that it has become a prominent strategy for the development of Iran’s health system [34]. To achieve our purpose, we will follow four objectives in our selected setting: to deeply understand the situation regarding underlying issues of quality of care; to explore the views of stakeholders about changes and initiatives that may be proposed in regard to the quality improvement; to define some changes to improve the quality of care; and to reflect on methods that are to be applied in order to realize the three previous objectives.

## Methods/design

The selection of our study methodology was guided by the idea of ‘complexity in healthcare organizations and how quality improvement can be approached in these settings’ [43,44]. Because we considered ‘CG implementation in an ED’ as a complex problem situation, we selected Soft Systems Methodology (SSM) as the guiding methodology in this study to achieve our purpose.

### Soft systems methodology

SSM is believed to be the most ‘theoretically-informed’ of the soft systems approaches [38]. Mingers and White suggested SSM as ‘the most widely used and practical application of systems thinking’ [45]. It has also been proposed as the best methodology to develop and implement interventions in different settings and levels of health systems [46,47].

SSM, first introduced by Peter Checkland in 1981, is an inquiring/learning cycle that is being operated in form of an action research [48]. SSM user enters a problem situation with a clear framework of ideas (F), and a selected methodology (M). The user reflects on involvement in the action research so as to provide findings relevant to the F, the M, and the area of concern (A) in the problem situation [49]. The developed form of SSM consists of four basic activities: understanding a problem situation, including its cultural issues and power relations; building some ‘purposeful activity models’ relevant to the perceived situation in order to more inquire into the situation; comparing the models with the perceived situation, through a structured process of debate, to achieve ‘accommodations’ among different stakeholders with different perspectives, and find ideas that can improve the situation; and defining/implementing ‘culturally feasible and systemically desirable’ changes to improve the situation [50].

SSM itself is a set of purposeful activities planned to be implemented in order to inquire into the problem situation and improve it; so, in a single study, it is possible to use SSM in both designing the study process—‘SSM (p)’—and investigating its content—‘SSM (c)’ [51]. Therefore, we will use SSM to design our study methodology and fulfill our objectives.

### Conceptual framework of the study

**Figure 1:**
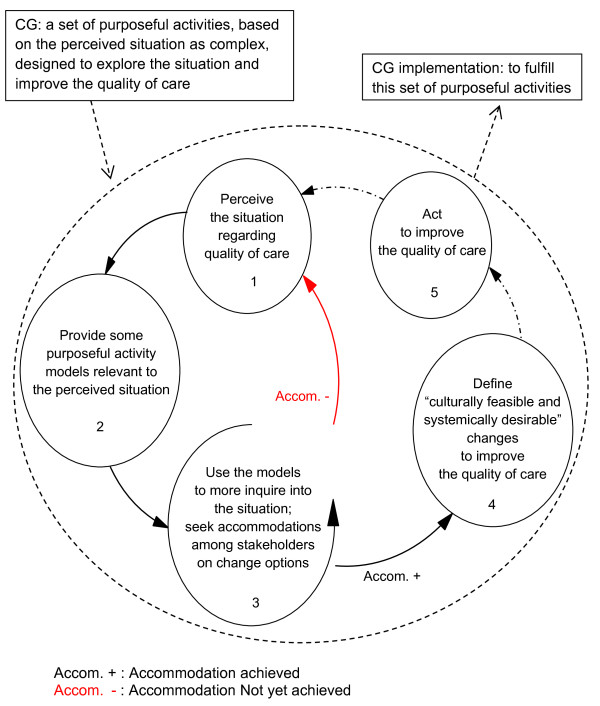
Clinical governance concept to be implemented in the selected setting.

We conceptualized CG as a cycle of SSM (Figure 1). We regarded CG as a set of purposeful activities, based on the perceived situation as complex, designed to explore the situation and improve the quality of care in our selected setting. This set of purposeful activities will build the steps of our action research methodology. Therefore, CG implementation will conceptually be to carry out that set of purposeful activities (*i.e.*, to accomplish our study methodology steps). We will be enabled to understand CG and its implementation by reflecting on involvement in this action research in our selected setting [49]. We will also consider the ‘quality of care’ as a concept constructed through interaction of stakeholders’ value judgments about different dimensions of care.

Our study has been started in accordance with the first step stated in Figure 1 in February 2012. It will be finished with ‘defining culturally feasible and systemically desirable changes to improve the quality of care’ through only one cycle of the action research (steps one to four in Figure 1) (See Additional file 1).

### Selecting an ED for conducting the research

Initially, we limited our choices to EDs of the general teaching hospitals affiliated with Tehran University of Medical Sciences (TUMS). Then we focused on three candidate hospitals that provide residency training in emergency medicine. AH interviewed with the director of the CG Department of TUMS to preliminarily assess these candidates. To do this, the interviewer asked questions about ‘enthusiasm of the managerial group for doing something to implement CG,’ ‘readiness of key people to initiate changes,’ ‘availability of resources,’ and ‘organizational climate regarding communication and staff cooperation.’ After reviewing the results of the interview, the research team prioritized the three EDs in a consensus meeting. Subsequently, AH presented a summary of the study steps to chief executives and directors of the EDs of the three hospitals. AH also assessed their readiness to adopt the study protocol through separate interviews. Finally, the research team discussed on the results of these interviews and selected one ED by consensus to conduct the study through the following steps.

### Step one: perceiving the situation regarding quality of care

SSM starts with exploring a problem situation by inquiring about key stakeholders, important issues, interconnected problems, behavioral patterns, cultural characteristics, and power relations in the situation. This exploration results in understanding of the problem situation [48,50]. Therefore, in this step of our research, we will use a variety of methods to collect the relevant data and understand the situation as described below.

### Interview with key stakeholders

We will conduct semi-structured interviews with key stakeholders regarding the quality of care. Interviewees will be selected purposefully from among the hospital’s chief executive and his assistants, the general manager, the matron, supervisor nurses, the director of the ED, attending physicians, emergency nurses, residents, patients, and the people who accompany patients in the ED. We estimate that there will be approximately 40 diverse interviews. Purposeful sampling of interviewees will be started with typical case sampling and continued with snowball sampling, considering the maximum diversity. To obtain a comprehensive account of the situation, we will also use other sampling strategies, such as ‘emergent sampling,’ selecting ‘confirming and disconfirming cases,’ and selecting ‘politically important cases’ [52]. All participants will be informed about the study objectives and methods (*e.g.*, digital recording of interviews), and our ethical policy of voluntary participation and participants’ confidentiality. They will be asked to fill a consent form prior to the interviews. The interviews will be conducted according to an interview guide at an agreed time and preferably in the hospital (see the interview guide in Additional file 2). It is estimated that each interview last about one hour. We will digitally record all of the interviews after participants’ permission. All recorded interviews will be downloaded, labeled, and transcribed verbatim immediately. We may also take some handwritten notes during interviews.

### Review of related documents

We will review all related documents that have been archived in the hospital during the previous two years. In this regard, all statistical reports, minutes of committee meetings, Directives, regulations, programs, guidelines, policies and procedures, and evaluation reports that are relevant to the quality of care in the ED will be included. We will start with convenience sampling of the documents and ask the key persons to continue with snowball sampling [52]. After obtaining permission from the holders, all of the selected documents will be reviewed at its archived location. We will take notes to extract the relevant data, with the guide of a sensitizing framework (see Additional file 3). The reviewers will also record their emergent interpretations during the review.

### Observation in hospital committee meetings

Consulting with the coordinator of the hospital committees, we will attend relevant committee meetings to observe how the committee members engage in issues regarding quality of care. We will initially focus on ‘accident and emergency services committee,’ ‘mortality and morbidity committee,’ ‘infection control committee,’ ‘internal audit committee,’ ‘ethics committee,’ and ‘clinical governance committee’ meetings that are to be held during the five-month period estimated for the first step of our study. The attending researcher will act as a ‘complete observer,’ who is sensitive to data concerning the participants’ pattern of interaction as well as their roles, norms, and values. Meeting discussions will be digitally recorded with permission. All of the recordings will be downloaded, labeled, and transcribed immediately. The attending researcher will also take short notes during the observation and complete them immediately after the meeting.

### Observation in the emergency department

We will observe how the healthcare providers in the ED will be involved in quality aspects of the care, if necessary, to confirm data gathered by the other methods described above. Therefore, the nature and amount of the previously collected data that need to be confirmed will determine the number of field observations and how these will be selected. We will be ready to select observation times from among all working shifts (morning, evening, and night) and also cover all ED areas, including ‘triage,’ ‘fast track,’ ‘critical care,’ ‘acute 1,’ and ‘acute 2’ areas. We will obtain permission for all observation sessions. The observer researcher will be a ‘complete observer’ who is sensitive to data regarding participants’ roles, norms, and values, as well as their interactions with each other, and with patients and accompanying persons. Each field observation session is estimated to last approximately two hours. The observer researcher will take short handwritten notes during the observations. He will also leave the field for short periods at intervals during the observations to record audio notes. These notes will be downloaded, labeled, transcribed, and compiled with handwritten notes to make the observation reports.

### Construction of meaning

Because SSM acknowledges socially constructed realities [40,48], we will use an iterative thematic analysis drawing on a constructivist grounded theory approach to analyze and interpret data [53]. All transcripts and handwritten notes will be read and coded independently by two researchers soon after the production. Closely related codes will be grouped into related categories. As the study continues, newer data, codes, and categories will be produced, compared constantly with the previous ones, and added to them. This process results in the categories to be modified and related into major emergent themes [53]. Codes, categories (subthemes), and themes will be discussed between the two researchers and verified by all other researchers.

As our focus of study directs our approach to data collection (*i.e.*, to design the interview guide, to set the sensitizing concepts for review of documents and observations, and to select data sources), it will also direct the analysis and interpretation of the collected data. Therefore, the emergent themes and subthemes will carry the notions of ‘quality-of-care issues’ and ‘relevant actions and processes that construct those issues’ in the selected ED.

Themes will then be interpreted and presented, along with subthemes and their interrelations, to shape the perception of the situation regarding quality of care. The presentation will be in both a narrative form and a ‘rich picture.’ Rich pictures are effective tools used to achieve shared understanding among participants and form the debate about the issues of the situation [54]. It is recommended that rich pictures should reflect the most arguable issues from the stakeholders’ points of view [48,50]. Therefore, during the study, we will repeatedly use a member-checking strategy to ensure that the emergent themes and the rich picture are in accordance with the participants’ ideas.

### Step two: providing relevant ‘purposeful activity models’

Checkland believes that, in any given problem situation, there are various stakeholders with different worldviews, interests, and purposes [40]. Grounded on these different individual properties that are unique to a particular situation, each stakeholder assumes performing a definite set of ‘purposeful activities’ will achieve a specific goal and also improve the situation [48]. In SSM, these notional sets of human purposeful activities are used to build ‘conceptual models’ that are ‘relevant’ to the situation [50]. Based on the perceived situation, SSM user should ‘select’ several relevant sets of human purposeful activities [48]. To build the conceptual models, each selected notion should be clearly described by a ‘root definition’ and a ‘CATWOE’^a^[48]. A ‘conceptual model’ is then built accordingly [48,50]. Conceptual models must have the characteristic properties of a ‘system’ (*i.e.*, hierarchical structure, emergent properties, communication, and control) [48,50]. Thus, each conceptual model should consist of some (usually five to nine) activities that are dependent upon each other, definition of measures of performance for the notional system, and monitor and control activities [48,50].

In this study, according to the above instructions, we will build some conceptual models. Since the themes and subthemes (that emerge in step one) represent ‘quality-of-care issues’ and ‘relevant actions and processes that construct those issues,’ we will insightfully use them to select and describe relevant sets of purposeful activities and build conceptual models. According to Checkland, ‘no human activity system is intrinsically relevant to any problem situation’ [48]; therefore, although our conceptual models will be grounded in real world data, these models will be just insightful propositions for improving the quality of care, which should be tested among the study participants. In this regard, to achieve the purpose of modeling (in the next step), we will try to build a number of conceptual models. Because we will be focused on CG for quality improvement in the selected ED, all of our conceptual models will be subsumed under the rubric of ‘the relevant models of CG.’

### Step three: seeking accommodations among stakeholders

Conceptual models built in SSM do not reflect actions of the real world [48]. In other words, activities contained in conceptual models are not to be executed identically in the real situation [48]. The models are only ‘intellectual devices’ used to further explore the situation, ‘surface’ different stakeholders’ interests and perspectives, seek accommodations among stakeholders, and learn about changes that can be made to improve the situation [50]. For this purpose, it is required to ‘compare’ conceptual models with the real situation through structured debates among stakeholders [48,50].

In this step of our study, consulting with the hospital’s chief executive and the director of the ED, we will select a diverse combination of the previously interviewed participants to hold focus group discussions. Our criteria for selection will require that the group members be well-informed about the situation. They must also be willing to cooperate and have no obstacle for participation in the group. After selecting the group members, we will hold a briefing session to make them familiar with the concepts of ‘rich picture,’ ‘purposeful activity system,’ ‘root definition,’ ‘CATWOE,’ and ‘conceptual model,’ and how we are going to use them. The briefing session will be held in the hospital, and is estimated to last about one hour. Then, we will schedule focus group meetings and invite partcipants accordingly. If it will not be possible for a patient to attend the meetings, one of the researchers will play his/her role instead. We will use a guide to conduct the meetings. Group discussions will be focused on comparing the ‘conceptual models’ (*i.e.*, the insightful propositions for improving the quality of care) with the ‘rich picture’ (*i.e.*, the perceived situation regarding quality of care). To carry out the comparison, we will present the rich picture and one of the conceptual models to the participants and start asking some questions: ‘Is the proposed conceptual model relevant to the situation?,’ ‘Do the activities of the conceptual model ever take place in the real situation?,’ ‘How are they performed?,’ ‘How do you—participants—judge those activities?,’ ‘What can be really done to improve the situation?’ In order to more explore the situation and clarify participants’ viewpoints, we will continue by asking follow-up and probing questions [50]. It is estimated that each meeting will last about two hours. All conversations will be digitally recorded by permission. Short notes will be taken during discussions and completed soon after the meetings. We will also download and transcribe the recorded files.

During the debates, the participants’ views on ‘quality-of-care issues’ and ‘changes that may improve the quality of care’ will be further explored. Therefore, based on the participants’ accommodations, it will become apparent whether a given conceptual model and its included activities are really ‘relevant’ to the situation and can lead to finding changes. If the debate does not reach to an accommodation among participants, we will propose other conceptual models (previously built or to be built) in subsequent meetings. To build new conceptual models, we will analyze the data collected from the meetings, as was stated above (in ‘construction of meaning’). Newer themes will be constructed by continuous comparing of the emergent themes with the original ones. This process can lead to the modification of the rich picture, the emergence of newer relevant systems, and the formation of newer conceptual models.

For the purpose of this step to be achieved, the mentioned cyclic process may be repeated several times [48,50]. Therefore, we cannot determine the number of focus group meetings. Nevertheless, based on the very complexity of the problem situation in this study, we think there will not be less than five or six meetings.

### Step four: defining changes to improve the quality of care

The final purpose of the focus group discussions is to find changes that, from the perspectives of the participants, are ‘desirable’ regarding improvement of the quality of care, and ‘feasible’ concerning the current culture. During the debates, the participants’ worldviews will be contrasted with each other, so that accommodations can be made on what will be a change to improve the quality of care. Changes may be about structures, processes, and attitudes [48,50]. As stated above, we will seek accommodations among stakeholders on which conceptual model and its included activities are really ‘relevant’ to the situation. Then, it will become possible to find changes through the process of ‘comparison.’ During this process, our criterion for defining changes is participants’ agreement with suggested change statements. We will then take those statements as defined changes to improve the quality of care in the selected ED.

### Reflecting on the study as a whole

As stated earlier, the overarching purpose of this study is to understand how CG can be defined and implemented in an ED. We considered ‘CG implementation in an ED’ as a complex problem situation. Therefore, assuming SSM as a useful methodology in approaching this kind of situations, we used it to design the process of our action research study and inquire into its content. We conceptualized CG as a set of purposeful activities. We assumed implementing that set of purposeful activities (*i.e.*, the conceptualized CG) as our study steps will yield two results: the quality situation of the selected ED will be explored; and the changes to improve the quality of care in the selected ED will be found.

During this action research, we will learn about CG and its implementation in the selected setting, through critical reflection on our assumptions [49]. In this regard, we will keep a descriptive journal of different aspects of the study, including how the sources of data will be selected; how data will be collected; what data will be collected; how data will be analyzed and interpreted; how conceptual models will be built; how conceptual models will be used; how focus group discussions will be conducted; how accommodations will be found; what influencing events will occur; and what contextual factors we will face. Reviewing the journal, we will also write analytic memos throughout the research process. We will use some sensitizing concepts to guide our memos, including complexity of quality improvement in the ED; accountability toward improving the quality of care in the ED; efficacy of SSM and the methods used in achieving our objectives; problems and obstacles to the implementation of the study steps; and modifications that need to be made to the study methods and processes. As memos develop over time, new hypotheses will be formed. This will lead to further thinking, searching for approving or disapproving data, and further memos [53]. Finally, putting the memos in the context, we will develop the emergent understanding of the whole study by narrative synthesis of the memos.

### Ethical considerations

Ethics approval for this study was obtained from the Ethics Committee of TUMS. In order to gain access to the setting, the research team will follow all necessary legal procedures. We will obtain the letter of introduction from the hospital’s chief executive and provide it to the relevant authorities. Researchers will respect all stakeholders and their right to voluntarily participate in the study. All participants will be informed about the objectives of each study step, methods of participation, methods of data collection (*e.g.*, digital recording of interviews and discussions), and protected confidentiality of the participants. We will ask them to fill a consent form prior to interviews and focus group discussions. We will also obtain necessary permissions for all field observation sessions.

## Discussion

In the late 1990s, the England’s Department of Health introduced ‘clinical governance’ as a framework to make healthcare organizations ‘accountable for continuously improving the quality of their services’ [21]. The new framework was based on the NHS specific history, context, and conditions [23]. At that time, considering the underlying issues, CG was conceptualized as the ‘seven pillars’ standing on the ‘five solid foundations’ [23]. In 2009, the implementation of CG became mandatory for all hospitals in Iran. However, Iranian hospitals work in a different context. Therefore, it is expected that these organizations will face different governance issues in their way toward quality improvement.

In spite of the assumed similarities in decision-making processes in different countries, it is advocated that each country should consider its specific underlying situation in planning for the quality improvement [55]. Implementation of CG is subject to the same condition [56]. CG is a complex, dynamic, and strategic framework that requires a comprehensive understanding of existing issues in order to identify and facilitate appropriate processes for being effectively implemented [57,58]. Taking these issues into account, it seems that the mere dissemination of the seven pillars of CG and some initiatives will not necessarily result in a successful CG implementation in Iranian hospitals. It is essential to develop comprehensive interventions to drive substantial changes [59,60].

In recent years, systems approaches have been more advocated in health areas due to the need for better understanding of influential factors in healthcare environment and overcoming health systems’ complexities [55,61-63]. But they are different in underlying assumptions, methodologies, and practical methods [38]. According to the ‘hard’ systems paradigm, the idea of CG can be captured in a model such as the ‘seven pillars’ model. Thus, CG implementation would focus on interventions that aim to strengthen the seven pillars in order to optimize ‘the system’ and improve the quality of care. In contrast, based on the ‘soft’ systems perspective, CG and quality improvement in a selected healthcare setting is a problem situation that requires to be explored in order to find relevant issues and appropriate interventions; thus, initially, no *a priori* model of CG is regarded as suitable for the given situation.

Using SSM as a problem structuring approach, we will do this action research as a vehicle to understand CG and how it can be implemented in the selected ED. We will record all events and research processes thoroughly, including how we will overcome obstacles in each study step. Thus, reflections on involvement in this study will make it possible to explain the following subjects in the selected ED: What issues need to be governed in order to improve the quality of care; what CG means; how CG can be implemented; and how SSM can be used to facilitate the implementation of CG.

It has been noted that description of contexts and operational challenges in quality improvement projects has a profound effect on the development of implementation science and the related initiatives [64]. Therefore, we intend to disseminate the results of this study focusing on ‘a thick description of contexts and complexities’ of quality improvement through CG implementation in the selected setting. The results can also be instructive for, and employed by, boards and executives governing other clinical settings to facilitate CG implementation.

This is also the first study in Iran’s health system that uses SSM as the guiding methodology. We anticipate that some issues regarding the organization’s culture (*e.g.*, teamwork, open communication, and power relations due to professionalism) will be among our study challenges. Nevertheless, the researchers’ experience in qualitative methods, along with flexible use of SSM and its relevant ‘intellectual devices’ will help us overcome those challenges. Moreover, we hold the support of the management team in the selected ED.

## Endnotes

^a^CATWOE is a mnemonic for Customers, Actors, Transformation process, Worldview, Owner(s), and Environmental constraints.

## Abbreviations

CG: Clinical Governance; ED: Emergency Department; MOHME: Ministry Of Health and Medical Education; SSM: Soft Systems Methodology; TUMS: Tehran University of Medical Sciences.

## Competing interests

The authors declare that they have no competing interests.

## Authors’ contributions

AH and MM conceived of the study. AH, MM, ABM and HR contributed to the study design and coordination. MS, MJ, DF, AK and ZA participated at various times in the study design and coordination, or were involved in selecting the field of study. AH drafted the manuscript. MM, ABM, HR, MS, MJ, DF, AK and ZA provided feedback for revision of the draft manuscript. All authors read and approved the final manuscript.

## Authors’ information

AH is a Medical Doctor with more than 10 years of experience as a top-ranked manager at district and provincial levels of Iran’s health system, and is now a PhD candidate at School of Health Management and Information Sciences (SHMIS), Tehran University of Medical Sciences (TUMS). AH is also a Clinical Governance Country Assessor, and a Member of the Scientific Committee at Office of Hospital Management and Clinical Excellence (OHMCE), Ministry Of Health and Medical Education (MOHME). MM is an Associate Professor at SHMIS, TUMS. ABM is an Assistant Professor at SHMIS, TUMS. HR is an Assistant Professor at SHMIS, TUMS, and the Director General for OHMCE, MOHME. MS is an Assistant Professor at Community Medicine Department, School of Medicine, TUMS, and also the Director of Clinical Governance Department at TUMS. MJ is an Assistant Professor in Internal Medicine (Nephrology) Department, School of Medicine, and the Chief Executive of Hazrat Rasoul Akram Hospital, TUMS. DF is an Assistant Professor in Emergency Medicine Department, School of Medicine, and the Director of Emergency Department at Hazrat Rasoul Akram Hospital, TUMS. AK is a Medical Doctor and currently a PhD candidate at SHMIS, TUMS. ZA is a PhD candidate at SHMIS, TUMS.

## Supplementary Material

Additional file 1 **Time table.** The predicted timetable for the research process. Click here for file

Additional file 2 **Interview guide.** The flexible question guide for conducting the semi structured interviews with the participants Click here for file

Additional file 3 **Document review framework.** The sensitizing framework used as a guide to extract relevant data from the written documents. Click here for file

## References

[B1] Donabedian A Evaluating the Quality of Medical Care Milbank Q 2005 83 4 691 729 reprinted article 10.1111/j.1468-0009.2005.00397.x 16279964 PMC2690293

[B2] Donabedian A The quality of care: How can it be assessed? Arch Pathol Lab Med 1997 121 11 1145 1150 9372740

[B3] Lohr KN Medicare: A Strategy for Quality Assurance 1990 National Academy Press, Washington D.C 25144047

[B4] Harteloh PP The Meaning of Quality in Health Care: A Conceptual Analysis Health Care Anal 2003 11 3 259 267 14708937 10.1023/B:HCAN.0000005497.53458.ef

[B5] World Health Organization Quality of care: a process for making strategic choices in health systems 2006 WHO, Geneva

[B6] Mitchell PH Hughes RG Defining Patient Safety and Quality Care Patient Safety and Quality: An Evidence-Based Handbook for Nurses 2008 Agency for Healthcare Research and Quality, Rockville, MD 21328752

[B7] Sizmur S Redding D Core Domains For Measuring Inpatients' Experience Of Care 2009 Picker Institute Europe [Available from: www.pickereurope.org]

[B8] Oermann MH Dillon SL Templin T Indicators of Quality of Care in Clinics: Patients' Perspectives J Healthc Qual 2000 22 6 9 12 10.1111/j.1945-1474.2000.tb00159.x 11186040

[B9] Blumenthal D Quality of Care - What Is It? N Engl J Med 1996 335 12 891 894 10.1056/NEJM199609193351213 8778612

[B10] Rogers PG RAID methodology: the NHS Clinical Governance Team’s approach to service improvement Clin Govern Int J 2006 11 1 69 80 10.1108/14777270610647047

[B11] Mugglestone M Maher L Manson N Baxter H Accelerating the improvement process Clin Govern Int J 2008 13 1 19 25 10.1108/14777270810850599

[B12] Powell A Rushmer R Davies H A systematic narrative review of quality improvement models in health care 2009 NHS Quality Improvement Scotland [http://www.healthcareimprovementscotland.org/previous_resources/hta_report/a_systematic_narrative_review.aspx]

[B13] Walshe K Pseudoinnovation: the development and spread of healthcare quality improvement methodologies Int J Qual Health Care 2009 21 3 153 159 10.1093/intqhc/mzp012 19383716

[B14] Wardhani V Utarini A van Dijk JP Post D Groothoff JW Determinants of quality management systems implementation in hospitals Health Policy 2009 89 3 239 251 10.1016/j.healthpol.2008.06.008 18752866

[B15] Solberg LI Kottke TE Brekke ML Magnan S Davidson G Calomeni CA Conn SA Amundson GM Nelson AF Failure of a continuous quality improvement intervention to increase the delivery of preventive services: a randomized trial Eff Clin Pract 2000 3 3 105 115 11182958

[B16] Berg M Schellekens W Bergen C Bridging the quality chasm: integrating professional and organizational approaches to quality Int J Qual Health Care 2005 17 1 75 82 10.1093/intqhc/mzi008 15668314

[B17] Øvretveit J Would it work for us? Learning from quality improvement in Europe and beyond Jt Comm J Qual Improv 1997 23 1 7 22 9116888 10.1016/s1070-3241(16)30290-5

[B18] Shaw CD Health-care quality is a global issue Clin Govern Bull 2002 3 2 2 8

[B19] WHO Regional Committee for the Eastern Mediterranean Improving hospital performance in the Eastern Mediterranean Region. Technical paper EM/RC56/5 2009 WHO Regional Office for the Eastern Mediterranean, Fez, Morocco [Available from: http://www.emro.who.int/rc56/documents.htm]

[B20] Department of Health The New NHS: modern and dependable 1997 The Stationery Office, London

[B21] Department of Health A First Class Service: Quality in the New NHS 1998 DOH, London

[B22] McSherry R Pearce P Clinical Governance: A Guide to Implementation for Healthcare Professionals 2011 3 Wiley-Blackwell, Chichester

[B23] Nicholls S Cullen R O'Neill S Halligan A Clinical governance: its origins and its foundations Br J Clin Govern 2000 5 3 172 178 10.1108/14777270010734055 11142803

[B24] Commission for Health Improvement [http://ratings2003.healthcarecommission.org.uk/ratings]

[B25] A guide to clinical governance reviews: mental health services 2003 [http://www.pdca.es/documentos/Guia_Revision_Governance_Salud_Mental.pdf]

[B26] Scally G Donaldson LJ Clinical governance and the drive for quality improvement in the new NHS in England BMJ 1998 317 7150 61 65 10.1136/bmj.317.7150.61 9651278 PMC1113460

[B27] Donaldson LJ Muir Gray JA Clinical governance: a quality duty for health organisations Qual Health Care 1998 7 suppl S37 S44 10339034

[B28] Office of Safety and Quality in Healthcare, Department of Health, Government of Western Australia Clinical governance[http://www.safetyandquality.health.wa.gov.au/initiatives/clinical_governance.cfm]

[B29] Wright L Malcolm L Barnett P Hendry C Clinical leadership and clinical governance: a review of developments in New Zealand and internationally 2001 [Available from: http://www.hiirc.org.nz/page/15339/clinical-leadership-and-clinical-governance/?show=popular&contentType=167&section=8959]

[B30] Pridmore JA Gammon J A comparative review of clinical governance arrangements in the UK Br J Nurs 2007 16 12 720 723 17851360 10.12968/bjon.2007.16.12.23723

[B31] The Islamic Republic of Iran’s Fourth Economic, Social and Cultural Development Plan Act 2004 (in Persian) [http://www.parliran.ir/index.aspx?siteid=1&pageid=2941]

[B32] The Islamic Republic of Iran’s Fifth Economic, Social and Cultural Development Plan Act 2011 (in Persian) [http://parliran.ir/index.aspx?siteid=1&siteid=1&pageid=3362]

[B33] The Islamic Republic of Iran’s Comprehensive Scientific Roadmap for Health 2010 (in Persian) [http://hbi.ir/info/banner/S&T_Map-Final.pdf]

[B34] The Islamic Republic of Iran's Health System Development Roadmap based on the Islamic-Iranian Model of Progress 2012 (in Persian) [http://siasat.behdasht.gov.ir/uploads/291_1041_Health%20Map%20Book%20esfand.pdf]

[B35] Mosadegh Rad AM A survey of total quality management in Iran: Barriers to successful implementation in health care organizations Leadersh Health Serv 2005 18 3 12 34 10.1108/13660750510611189 16167653

[B36] Manouchehri Moghadam J Ibrahimipour H Sari Akbari A Farahbakhsh M Khoshgoftar Z Study of patient complaints reported over 30months at a large heart centre in Tehran Qual Saf Health Care 2010 19 5 1 5 10.1136/qshc.2009.033654 20547707

[B37] Office of Hospital Management and Clinical Excellence An introduction to essentials of clinical governance (in Persian) 2011 Ministry of Health and Medical Education, Tehran, Iran

[B38] Jackson MC Systems Thinking: Creative Holism for Managers 2003 John Wiley & Sons, Ltd, Chichester, England

[B39] Reynolds M Holwell S Systems Approaches to Managing Change: A Practical Guide 2010 Springer (in association with Open University), London

[B40] Checkland P Systems Thinking, Systems Practice: A 30-year Retrospective 1999 John Wiley & Sons, Ltd, Chichester, England

[B41] Committee on the Future of Emergency Care in the United States Health System Hospital-Based Emergency Care: At the Breaking Point 2007 The National Academies Press, Washington, DC [Available from: http://www.nap.edu/catalog/11621.html]

[B42] Jensen KB Kirkpatrick DG The Hospital Executive's Guide to Emergency Department Management 2010 HCPro, Inc., Marblehead, MA

[B43] Berwick DM The science of improvement JAMA 2008 299 10 Reprinted 1182 1184 18334694 10.1001/jama.299.10.1182

[B44] Øvretveit J Producing useful research about quality improvement Int J Health Care Qual Assur 2002 15 7 294 302 10.1108/09526860210448465 12500654

[B45] Minger J White L A review of the recent contribution of systems thinking to operational research and management science Eur J Oper Res 2010 207 3 1147 1161 10.1016/j.ejor.2009.12.019

[B46] Kalim K Carson E Cramp D An illustration of whole systems thinking Health Serv Manage Res 2006 19 174 185 10.1258/095148406777888116 16848958

[B47] Braithwaite J Hindle D Iedema R Westbrook JI Introducing soft systems methodology plus (SSM+): why we need it and what it can contribute Aust Health Rev 2002 25 2 191 198 10.1071/AH020191 12046149

[B48] Checkland P Scholes J Soft Systems Methodology in Action: A 30-year Retrospective 1999 John Wiley & Sons, Ltd, Chichester, England

[B49] Checkland P Holwell S Action Research: Its Nature and Validity Syst Pract Action Res 1998 11 1 9 21 10.1023/A:1022908820784

[B50] Checkland P Poulter J Reynolds M, Holwell S Soft Systems Methodology Systems Approaches to Managing Change: A Practical Guide 2010 Springer, London 191 242

[B51] Checkland P Winter M Process and content: two ways of using SSM J Oper Res Soc 2006 57 12 1435 1441 10.1057/palgrave.jors.2602118

[B52] Patton MQ Qualitative research and evaluation methods 2002 3 Sage, Thousand Oaks, CA

[B53] Charmaz K Constructing Grounded Theory: a practical guide through qualitative analysis 2006 Sage Publications Ltd., London

[B54] Bell S Morse S Rich Pictures: a means to explore the ‘Sustainable Group Mind.’ The 16th annual international sustainable development research conference: 30 May - 01 Jun 2010; Hong Kong, China 2010 [Available from: http://oro.open.ac.uk/24617/]

[B55] World Health Organization Everybody business: strengthening health systems to improve health outcomes: WHO’s framework for action 2007 WHO, Geneva

[B56] Whitty PM Prescribing how NHS trusts ‘do’ quality: a recipe for committees but little action? Qual Saf Health Care 2004 13 5 328 10.1136/qshc.2004.011767 15465933 PMC1743900

[B57] Øvretveit J Evaluation informed management and clinical governance Br J Clin Govern 1999 4 3 103 109 10.1108/14664109910309683

[B58] Maddock A Kralik D Smith J Clinical Governance improvement initiatives in community nursing Clin Govern Int J 2006 11 3 198 212 10.1108/14777270610683137

[B59] Halligan A Donaldson L Implementing clinical governance: turning vision into reality BMJ 2001 322 1413 1417 10.1136/bmj.322.7299.1413 11397753 PMC1120478

[B60] Grol RP Bosch MC Hulscher ME Eccles MP Wensing M Planning and studying improvement in patient care: the use of theoretical perspectives Milbank Q 2007 85 1 93 138 10.1111/j.1468-0009.2007.00478.x 17319808 PMC2690312

[B61] World Health Organization The World health report 2000: health systems: improving performance 2000 WHO, Geneva

[B62] de Savigny D Adam T Systems thinking for health systems strengthening 2009 WHO, Geneva

[B63] Alliance for Health Policy and Systems Research Strengthening health systems: the role and promise of policy and systems research 2004 Global Forum for Health Research, Geneva

[B64] Øvretveit J Understanding the conditions for Improvement: research to discover which context influences affect improvement success BMJ Qual Saf 2011 20 Suppl 1 i18 i23 10.1136/bmjqs.2010.045955 PMC3066695 21450764

