# Supplementary material for: Clinical governance implementation in a selected teaching emergency department: a systems approach
Source: Implement Sci. 2012 Sep 10;7:84. doi: 10.1186/1748-5908-7-84 (PMC3457909; doi:10.1186/1748-5908-7-84)
Supplement: Additional file 1 — Time table. The predicted timetable for the research process. [file 1748-5908-7-84-S1.pdf]

Additional file 1: Time table. The predicted time table for the research process

Activity / Year/Month	2012											2013
	2	3	4	5	6	7	8	9	10	11	12	1
Selecting an emergency department for conducting the research	•											
Perceiving the situation regarding quality of care		•	•	•	•	•						
Providing relevant "purposeful activity models"						•	•					
Seeking accommodations among stakeholders							•	•	•	•	•	
Defining changes to improve the quality of care									•	•	•	
Writing final report											•	•
