# Supplementary material for: Clinical governance implementation in a selected teaching emergency department: a systems approach
Source: Implement Sci. 2012 Sep 10;7:84. doi: 10.1186/1748-5908-7-84 (PMC3457909; doi:10.1186/1748-5908-7-84)
Supplement: Additional file 2 — Interview guide. The flexible question guide for conducting the semi structured interviews with the participants [file 1748-5908-7-84-S2.pdf]

Additional file 2: Interview guide. The flexible question guide for conducting the semi structured interviews with the participants

A) The interviewer provides a summary of study objectives and methods.

B) The interviewer clarifies the ethical policy of voluntary participation and participants' confidentiality.

C) The interviewer asks for permission to record the interview (consent to record).

D) Questions: (use questions as a guide only)

1. In your opinion, what are the characteristics of a high quality emergency department?
   - Probe: Can you explain more, please? (to further clarify the characteristics)
   - Probe: Can you give an example, please?
2. How is the quality of care in this emergency department?
   - Follow: Why do you think the quality situation is like this?
   - Probe: Can you explain more about the quality problems, please?
3. Why do the quality problems remain unsolved?
   - Follow: What are the main obstacles to improving the quality of care in this emergency department?
   - Probe: Can you explain more, please?
   - Probe: Can you give an example, please?
4. How do different people or groups relevant to the quality problems act in this emergency department?
   - Follow: How do those people/groups lose or gain from the situation?
   - Follow: What is those people/groups' position on addressing the quality problems?
   - Follow: What resources do those people/groups have to influence the situation?
5. Who is accountable for the quality of care and its improvement in this emergency department?
   - Follow: How does he/she perform in that position?
   - Follow: What authority or other resources does he/she have in his/her position?
6. In your opinion, what can be done in this emergency department in order to improve the quality of care?
7. Is there anything else you would like to add?
