# Supplementary material for: Clinical governance implementation in a selected teaching emergency department: a systems approach
Source: Implement Sci. 2012 Sep 10;7:84. doi: 10.1186/1748-5908-7-84 (PMC3457909; doi:10.1186/1748-5908-7-84)
Supplement: Additional file 3 — Document review framework. The sensitizing framework used as a guide to extract relevant data from the written documents. [file 1748-5908-7-84-S3.pdf]

Additional file 3: Document review framework. The sensitizing framework used as a guide to extract relevant data from the written documents

A) Issues regarding the quality of care in the emergency department and their causes

B) People/groups that are/should be involved in addressing the quality issues in the emergency department

C) How different people/groups communicate for raising the quality issues in the emergency department and finding ways to improve the quality of care

D) Initiatives/interventions proposed/executed in order to improve the quality of care in the emergency department

E) Repeated and/or chronic quality problems in the emergency department

F) Confirming and/or disconfirming evidence to findings derived from the interviews and the observations

G) Issues that need to be confirmed/disconfirmed through subsequent interviews and/or observations
